# Is *Betula carpatica* genetically distinctive? A morphometric, cytometric and molecular study of birches in the Bohemian Massif with a focus on Carpathian birch

**DOI:** 10.1371/journal.pone.0224387

**Published:** 2019-10-31

**Authors:** Ivan Kuneš, Rostislav Linda, Tomáš Fér, Petr Karlík, Martin Baláš, Jana Ešnerová, Jan Vítámvás, Jan Bílý, Tomáš Urfus

**Affiliations:** 1 Department of Silviculture, Faculty of Forestry and Wood Sciences, Czech University of Life Sciences Prague, Czech Republic; 2 Department of Botany, Faculty of Science, Charles University, Prague, Czech Republic; 3 Department of Forest Ecology, Faculty of Forestry and Wood Sciences, Czech University of Life Sciences Prague, Czech Republic; 4 EXTEMIT-K, Faculty of Forestry and Wood Sciences, Czech University of Life Sciences Prague, Czech Republic; Institute for Biological Research, SERBIA

## Abstract

Until recently, Czech taxonomists often treated *Betula carpatica* as a distinct species. Several morphological traits for distinguishing *B*. *carpatica* from *B*. *pubescens* or other birches are described in literature; however, it has been proven impossible to reliably identify *B*. *carpatica* in the field. With the use of morphological and molecular approaches, we intended to assess the position of *B*. *carpatica* in the context of other birch taxa reported from the Bohemian Massif and to find more reliable morphological traits for their identification. In our dataset, we distinguished the following birch taxa referred to in the recent Czech literature: *B*. *pendula*, *B*. *pubescens*, *B*. *carpatica*, *B*. *oycoviensis*, *B*. *nana*, *B*. *petraea* and *B*. ×*seideliana*. We complemented them with triploids and several diploid and tetraploid “working units” into which we included intermediate individuals that in terms of morphology did not unambiguously match any of the abovementioned birch taxa. Holoploid genome size was measured to determine the ploidy level. To identify genetic relationships between selected taxa and “working units”, microsatellite analyses were performed. Model-based STRUCTURE analysis together with principal coordinates analysis (PCoA) based on genetic distances was performed to identify the similarities in multilocus genotype data between groups distinguished in the dataset. The applied analyses were not able clearly to distinguish any group among tetraploid individuals. In this light, it was of no use to search for any more reliable morphological traits of *B*. *carpatica* and also *B*. *petraea*. Among diploids, *B*. *nana* was always distinguished, in contrast to *B*. *oycoviensis*, which was not genetically recognized despite being usually morphologically distinct. Based on our results and a literature review, we suggest that *B*. *carpatica* and also the closely similar *B*. *petraea* should not be considered separate species. A similar conclusion seems relevant also for *B*. *oycoviensis*; however, further verification is desirable in this case.

## Introduction

Birches (ord. *Fagales*—fam. *Betulaceae*) are deciduous trees or shrubs commonly distributed across the north temperate zone [1–3]. The age of the genus *Betula* L. has been estimated to be ~60–75 Ma [2, 4] and the genus further diversified in the Oligocene era [4]. The taxonomy of the genus *Betula* is generally considered problematic [3, 5, 6] and the positions of several subordinated taxa are still ambiguous [7]. Genetic studies on birches (or on the whole subfamily Betuloidae) have lagged behind those of other plants [8]. According to Ashburner & McAllister [9], there are in total ca 64 birch species currently described. Nevertheless, various authors present different numbers, usually ranging between 30 and 60 species [1, 10].

Variation in some birches is caused at least partly by ecotypic variability, particularly in response to climatic factors [11]. Uncertainties surrounding the identification of many birch taxa are probably caused also by polyploidy [12] as well as hybridization and introgression (back-crossing of hybrids with parental individuals) [3, 11], which is quite frequent in some birches and regions [13–15].

The commonly accepted basic chromosome number (meiotically reduced /haplophasic/ chromosome base number) of birches is x = 14, although some authors suggested a lower value of x = 7 [16]. Birches can form diploids (e.g. *B*. *pendula* Roth, *B*. *nana* L.), tetraploids (e.g. *B*. *pubescens* Ehrh.), pentaploids, hexaploids and octoploids [1], and there are also taxa with even more chromosome sets [17].

As stated above, hybridization is often mentioned as one of the important sources of uncertainty in the determination and taxonomic treatment of birches [3]. On the other hand, for example for the most common Central European birches *B*. *pendula* and *B*. *pubescens*, there may be many barriers to interspecific crossing in the wild [18], although their hybridizing is possible [19] and at least in some areas occurs occasionally [20, 21].

**Fig 1:**
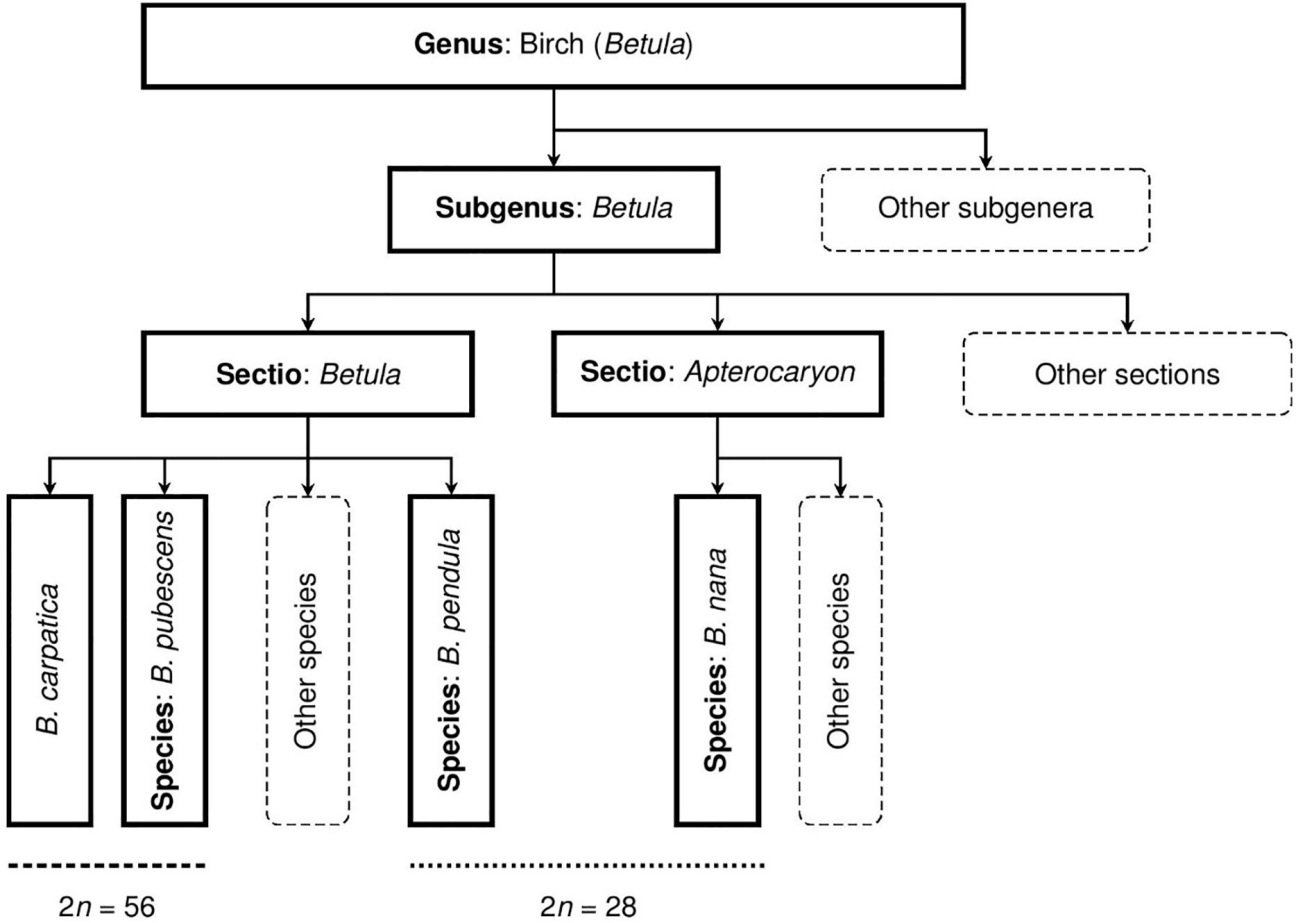
Taxonomic treatment of common birch taxa occurring in Central Europe according to Ashburner & McAllister [9]. Modified by R. Linda (suggested position of *B*. *carpatica*). The taxonomic rank is not identified in the case of *B*. *carpatica* because of its problematic determination. The respective ploidy levels of the taxa are indicated at the bottom of the diagram.

The systematics of *Betula* have been developing over time and various authors treat the genus differently [18]. Recently, Ashburner & McAllister [9] divided the genus *Betula* into four subgenera and these further into eight sections. In Central Europe, the majority of naturally occurring birch species belong to the section *Betula* (*B*. *pendula* Roth, *B*. *pubescens* Ehrh.) or the section *Apterocaryon* (*B*. *nana* L.), see Fig 1.

Diploid birches *B*. *pendula*, *B*. *nana* are reported to show less variation in morphological and molecular criteria than tetraploids [6, 11]. Nonetheless, next to the generally accepted diploid species *B*. *pendula* and *B*. *nana*, some local birch taxa (e.g. *B*. *oycoviensis* Besser, *B*. *atrata* Domin, *B*. *obscura* Kotula ex Fiek) are recognized in the Czech Republic and elsewhere in Central Europe, but their systematic status remains unresolved [9, 22, 23].

The systematics of tetraploid birches and hybrids are even more complicated. This problem pertains also to tetraploid *B*. *carpatica* Waldst. et Kit. ex Willd. (syn. *B*. *odorata* Bechst., *B pubescens* subsp. *odorata* (Bechst.) E. F. Warburg, *B*. *coriacea* Gunnarsson, *B*. *muritbii* Gaudin), see Walters [24]. In general, some birch taxa are variously treated in the taxonomic literature and their systematics include a full spectrum of taxonomic ranks with a number of described forms, varieties, subspecies and hybrids [25].

This study focuses on the question of the systematic status of *B*. *carpatica* in the Bohemian Massif. We decided to address this problem in the context of other birch taxa reported in the region. Next to *B*. *carpatica* and its broadly accepted cogeners (*B*. *pendula*, *B*. *pubescens* and *B*. *nana*), we included also *B*. *petraea* sensu Sýkora [26], *B*. *oycoviensis* Besser (from the only confirmed Czech population near the village of Volyně close to the town of Výsluní, “Volyně u Výsluní” for further reference), *Betula atrata* sensu Domin [27] and some triploid individuals in our study.

This approach enabled us to confront the level of genetic and morphometric divergence of *B*. *carpatica* from broadly accepted species (*B*. *pendula*, *B*. *pubescens* and *B*. *nana*) as well as from some hybrids and taxa whose taxonomic ranking is questionable or has not been resolved yet (*B*. *oycoviensis* and *B*. *petraea*).

As is the case of many birches, various publications are inconsistent in the taxonomic treatment of *B*. *carpatica*. *B*. *carpatica* was often referred to as a species in the Czech literature, even though changes to classification of *B*. *carpatica* were proposed very recently by Vašut [28]. Elsewhere during the post-war period, the treatment of *B*. *carpatica* as a species was advocated, for example, by Natho [29] and Szafer et al. [30].

Pawlowska [31] suggested that *B*. *carpatica* could be a hybridogenous species between *B*. *tortuosa* Ledeb. and *B*. *pubescens*. In her later work (on the basis of biochemical characters and chromosome counts), she proposed *B*. *pubescens* subsp. *tortuosa* var. *carpatica* [32]. Many authors and floristic books distinguished or reported *B*. *carpatica* as a subspecies of *B*. *pubescens* [24, 33–35]. The taxonomic treatment of *B*. *carpatica* as a subspecies of *B*. *pubescens* is included also in the recent Austrian, Slovak and German taxonomic literature [36–38]. Also the latest Polish botanical key [39] has inclined towards ranking *B*. *carpatica* a subspecies of *B*. *pubescens*. Ashburner & McAllister [9] include *B*. *carpatica* under *B*. *pubescens* var. *pubescens*. Still, other studies do not mention *B*. *carpatica* at all [12, 40, 41].

As mentioned above, the principal literature sources on the flora of the Czech Republic considered *B*. *carpatica* until very recently a species [42, 43]. The foresters usually respected the existing taxonomy used in the Czech Republic [42, 43]. They theoretically distinguished *B*. *carpatica* from *B*. *pubescens* and suggested that applicability of *B*. *pubescens* in the mountains of Northern Bohemia is up to 1,000 m a.s.l. whereas *B*. *carpatica* should be used to restore forests above 1,000 m a.s.l. [44]. It is worth mentioning that the recommendations regarding the use of *B*. *carpatica* have also been newly implemented the latest Czech legislation (Ministerial Decree no. 298/2018, date of entry into force 01/01/2019). In fact, however, the locality and elevation served as criteria for classifying tetraploid individuals as either *B*. *pubescens* or *B*. *carpatica*. To obey the recommendations regarding the use of *B*. *carpatica*, we tried to determine the species more exactly to ensure relevant seed sources for the highest and environmentally most exposed sites of the air-polluted mountains in the northern part of the Czech Republic. However, using the traits commonly described in the literature [42, 43, 45], we were not able to reliably distinguish *B*. *carpatica* from *B*. *pubescens*. This inspired a study on the taxonomy of selected birch species [46], whose outcomes are summarised in the present study.

The questions raised at the beginning of our study were:

- Are we able to distinguish *Betula carpatica* within the complex of tetraploid birches as a species using morphology, genome size and microsatellite analysis?
- In general, are we able to distinguish more taxa of birches within the complexes of *B*. *pendula* and *B*. *pubescens* in the Bohemian Massif?
- Are the tetraploid birches reported in the Bohemian Massif of autopolyploid or allopolyploid origin?
- What is the position of the minor birch taxa under assessment among broadly accepted species of birch, including *B*. *nana*, in the Bohemian Massif?

The answers to these questions should help us to evaluate the position of *B*. *carpatica* and compare this taxon with other taxa of the genus *Betula* reported from the Bohemian Massif.

## Materials and methods

### Birch taxa included in the study

In addition to *Betula carpatica*, the following birch taxa were included in our study: *Betula pubescens*, *B*. *petraea*, *B*. *pendula*, *B*. *nana*, *B*. *atrata*, *B*. *oycoviensis* and *B*. ×*seideliana* Missbach.

### Sampling sites

**Fig 2:**
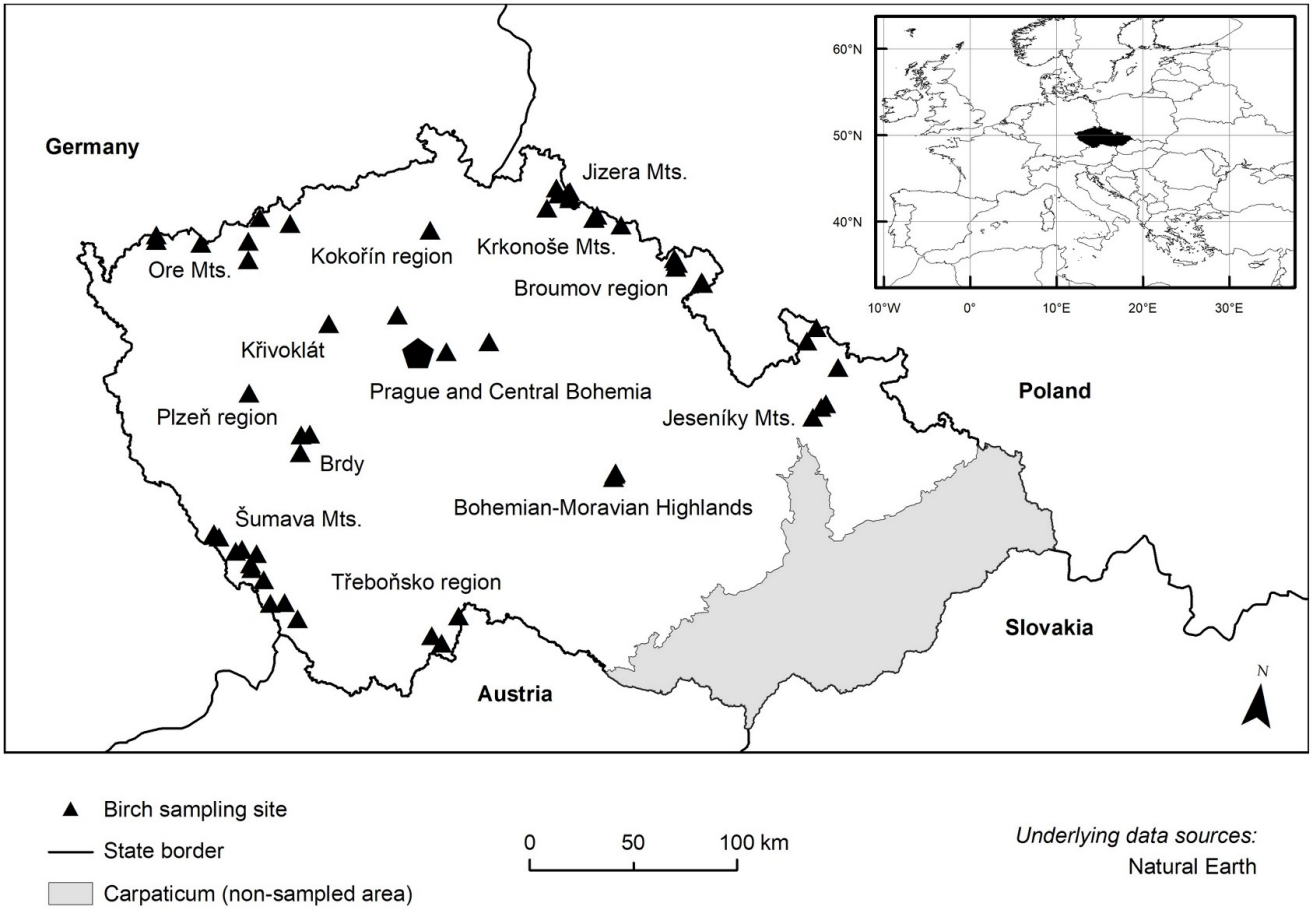
Distribution of 56 sampling sites of birch populations within the Bohemian Massif (Czech Republic).

Sampling was conducted at 56 localities in 13 regions covering 31 various habitats sensu Chytrý et al. [47] and 51 forest type complexes sensu Viewegh et al. [48] situated from lowlands to high mountain elevations, see Fig 2 and Table 1. A list of sampling localities detailing their general characteristics and their habitat as well as forest site classification is included in S1 Table.

**Table 1:** Numbers of analysed individuals of *Betula* spp. by the sampling region within the Bohemian Massif, locality and ploidy level. See S1 Table for the detailed characteristics of the sample localities.

Region	Sampled locality (local geografic names)	GPS coordintes of the sampled locality		Mean altitude	Number of analysed trees according to the ploidy level			
		Coord. system: WGS84		m a.s.l.	2n = 28	2n = 42	2n = 56	Σ
Brdy	Padrťské rybníky	N 49.6450°	E 013.7667°	640	0	0	6	6
	Třemšín	N 49.5683°	E 013.7783°	780	2	0	4	6
	Malá Praha	N 49.6540°	E 013.8217°	820	0	0	6	6
Broumov region	Čáp	N 50.5783°	E 016.1283°	700	5	0	0	5
	Koruna	N 50.5217°	E 016.3233°	750	2	0	2	4
	Pod Korunou	N 50.5267°	E 016.3150°	730	1	0	3	4
	Teplická Ozvěna	N 50.5950°	E 016.1383°	560	4	0	7	11
	Vlčí rokle	N 50.6027°	E 016.1215°	550	3	0	8	11
	Havraní Město	N 50.6143°	E 016.1127°	515	0	0	1	1
Bohemian-Moravian highlands	alej u Radostína	N 49.6535°	E 015.8813°	620	1	0	1	2
	Radostínské rašeliniště	N 49.6600°	E 015.8850°	620	2	0	7	9
	Velké Dářko	N 49.6400°	E 015.8750°	620	0	0	6	6
Hrubý Jeseník and its foothill area	Hrouda u Hor. Heřmanovic	N 50.3817°	E 017.1233°	280	2	0	2	4
	Malá kotlina	N 50.0400°	E 017.2100°	1240	3	0	3	6
	Rejvíz (east and west)	N 50.2200°	E 017.2967°	760	2	0	4	6
	Skorošice	N 50.3200°	E 017.0683°	380	7	0	0	7
	Skřítek	N 49.9933°	E 017.1600°	840	0	0	4	4
	Velká kotlina	N 50.0567°	E 017.2383°	1225	0	0	6	6
Jizerské hory Mts. (Jizera Mts.)	Čínská cesta	N 50.8583°	E 015.2533°	765	5	0	0	5
	Jizera	N 50.8333°	E 015.2733°	1010	3	0	0	3
	Lasičí (research plot)	N 50.8287°	E 015.3543°	980	5	0	0	5
	Malá Strana	N 50.7650°	E 015.2050°	715	3	0	1	4
	Panelka (research plot)	N 50.8188°	E 015.3523°	855	0	0	5	5
	Pytlácké kameny	N 50.8430°	E 015.3345°	960	3	0	0	3
	Velká Jizerská louka	N 50.8500°	E 015.3467°	830	0	0	8	8
Kokořín region	Hvězda	N 50.6038°	E 014.4353°	365	0	0	5	5
Krkonoše Mts. Giant Mts.)	Labský důl	N 50.7617°	E 015.5533°	1040	0	0	31	31
	Malá Kotelní jáma	N 50.7467°	E 015.5333°	1125	0	0	6	6
	Obří důl	N 50.7317°	E 015.7233°	1100	0	0	7	7
	Velká Kotelní jáma	N 50.7483°	E 015.5367°	1115	0	0	19	19
Krušné hory Mts. (Ore Mts.)	Boží Dar a Božídarský Špičák	N 50.4033°	E 012.9017°	1015	2	0	20	22
	Jezerka	N 50.5467°	E 013.4817°	635	9	0	0	9
	Novodomské rašeliniště	N 50.5517°	E 013.2700°	820	2	0	4	6
	Přebuz	N 50.3832°	E 012.5995°	890	0	0	2	2
	Úhošť	N 50.3633°	E 013.2383°	540	8	0	0	8
	Velké Jeřábí jezero	N 50.4068°	E 012.5940°	945	0	0	2	2
	Volyně u Výsluní	N 50.4425°	E 013.2185°	715	25	1	0	26
Křivoklát region	Leontýnský potok	N 50.1402°	E 013.8418°	412	4	0	3	7
Plzeň region	Kamenný rybník	N 49.7917°	E 013.3783°	340	0	0	9	9
Střední Polabí and Dolní Povltaví–Central Bohemia	Klánovice (Prague District)	N 50.0908°	E 014.6550°	250	1	0	8	9
	Kersko	N 50.1565°	E 014.9293°	194	3	0	10	13
	Minice	N 50.2198°	E 014.2933°	184	3	0	0	3
Šumava Mts. (Bohemian forest)	Horská Kvilda	N 49.0583°	E 013.5600°	1055	0	0	10	10
	Chalupská slať	N 48.9983°	E 013.6600°	905	0	0	11	11
	Jezerní slať	N 49.0383°	E 013.5717°	1070	1	1	13	15
	Kaňon Křemelné	N 49.1033°	E 013.4483°	745	1	0	7	8
	Novohůrecké slatě	N 49.1533°	E 013.3250°	875	0	0	5	5
	Obří hrad (Šafářův vršek)	N 49.1067°	E 013.5867°	805	1	0	5	6
	Paštěcké skály a bývalé Paště	N 49.1148°	E 013.4880°	670	8	1	5	14
	Slatinný potok a Gerlova huť	N 49.1650°	E 013.2883°	950	1	0	4	5
	Soumarský most	N 48.9133°	E 013.8183°	750	0	0	8	8
	Splavské rašeliniště (Strážný)	N 48.9017°	E 013.7267°	810	0	0	6	6
	Vltavský luh-Pěkná	N 48.8517°	E 013.9183°	730	0	0	7	7
Třeboň region	Červené blato	N 48.8587°	E 014.8080°	483	3	1	6	10
	Rašeliniště Pele	N 48.9592°	E 014.9655°	469	1	0	0	1
	Žofinka	N 48.8323°	E 014.8773°	474	4	0	1	5
**Sum**					**130**	**4**	**298**	**432**

We included in our study regions and localities of the Bohemian Massif from which the abovementioned birches have been reported in the literature, sites recommended by local authorities and locations listed in databases on the Czech flora (www.florabase.cz, www.pladias.org). One sample (*B*. *×seideliana*) was provided to us (see below). Sites with “low to zero commercial forestry management” (e.g. natural reserves and strictly protected zones of national parks or protected landscape areas) were preferred to ensure the sampling of local naturally-regenerated birch populations.

### Sampling

Sampling was realized between 2010 and 2018. If possible, we recorded the GPS position of each sampled tree and preliminarily determined its ploidy level and taxon (verification followed later after analysis by flow cytometry). We measured the tree height and stem diameter of each sampled birch and usually took three (ca 15–20 cm long) branchlets containing at least 10 leaves. The branchlets were sealed in a plastic bag with a water-soaked cotton pad and stored in the cold. Two of the sampled branches were used for morphological measurements, and the third branch was rush-shipped (within 3–5 days) to a laboratory so that its ploidy level could be determined by flow cytometry and that the remaining material could be subjected to analyses by molecular methods. After the laboratory verification of the ploidy level, each sampled birch was also “definitely” included in a particular birch taxon or “working unit”. See the *Distinguishing and classification of sampled birches* section later in the text.

Although the sampling on sites of reported occurrence of *B*. *carpatica*, *B*. *pubescens* or *B*. *petraea* was primarily focused on tetraploid taxa, some diploid birches were sampled (if found) on the surveyed sites for comparison. This approach increased the probability of sampling individuals with various ancestries, which are required for genetic analyses.

Out of the 432 birch individuals analysed, there were 130 diploid, 4 triploid and 298 tetraploid individuals (Table 1). The greater number of tetraploids compared to diploids is caused by the selection of sampling sites, as focused on sampling tetraploid individuals (the study chiefly concerns *B*. *carpatica*) to capture as much of their variability as possible.

### Comparative morphological analysis of leaves

The comparative morphological analysis of leaves was included in our study to provide quick general information on the morphological variability of all the birch taxa under assessment. The inherent limitations of this approach stem from the necessity to include only such foliar traits that can be measured for all birch taxa considered in our study. This is given by the requirements of the statistical methods.

The traits assessed in our study are as follows: blade length (mm), blade width (mm), leaf tip angle (°), petiole length (mm), distance of the widest part of a blade from the blade base (mm), number of leaf veins, blade width in the upper 1/4 (mm), distance from the leaf base to the 1^st^ tooth (mm) and 1^st^ vein angle (°). The abovementioned traits were determined, if possible, on four leaves from two branchlets (2 + 2) per each sampled tree. The calculated trait means of these four measurements then represented the sampled individual in further morphological analyses. The methods of measurement of the morphological traits and their analyses were described in detail, for example, by Linda et al. [49].

### Flow cytometry

The samples were analysed with propidium iodide flow cytometry method [50] to determine the ploidy level of each individual. As the internal standards, *Glycine max* (L.) Merril, small seed strain with 2C = 2.37 pg [51] and later *Solanum pseudocapsicum* L. with 2C = 2.61 pg were used. Calibration measurements of both the standards were done to enable comparisons between results obtained with the two internal standards. Two to three leaf stalks or (in case of *B*. *nana*) about 1.5 cm^2^ of leaf material (leaf stalks and blades) together with ca 1.8 cm^2^ of one of the internal standards was chopped in 0.5 ml of Otto I buffer [52]. The suspension was filtered through a 42-μm nylon mesh and incubated for ca 20 min at room temperature. Subsequently, the suspension was stained by a solution containing 1 ml of Otto II buffer [52], β-mercaptoethanol, propidium iodide and RNase IIA. Flow cytometry was performed using a Partec CyFlow flow cytometer (Partec GmbH, Germany) with a green solid-state laser (Cobolt Samba, 532 nm, 100 mW).

Information about ploidy was used to more precise distinguish the affiliation of sampled individuals to taxa and to determine the correct number of present alleles during the detection of microsatellite length. From the holoploid genome size, the 1Cx-value was established sensu Greilhuber et al. [53], that is, the DNA content of one non-replicated monoploid genome with a chromosome number of x = 14, to compare the chosen birch taxa (*B*. *pendula*, *B*. *oycoviensis*, *B*. *nana*, *B*. *pubescens*, *B*. *carpatica* and *B*. *petraea*) irrespective of ploidy at the level of basic genome size. Flow cytometry analyses were performed at the Department of Botany of the Faculty of Science at Charles University in Prague.

### Distinguishing and classification of sampled birches

The detailed assessment of sampled birches included the morphological evaluation of foliage, branching and habitus as well as an appraisal of their location and habitat.

As result of this assessment using identification traits and places of occurrence reported in the literature [26, 43, 45, 54, 55], and after analysis by flow cytometry, we distinguished the following taxa and “working units”:

(1) ***Betula pendula***: typical diploid representatives of the species.
(2) **Atypical *B*. *pendula***: diploid individuals showing the majority of morphological traits indicative of *B*. *pendula*, having, however, one or a few morphological features that are not common for the species (e.g. atypical shape of leaves, colour or structure of bark, absence of pendulous branchlets, etc.). These atypical traits assessed together, nonetheless, did not suggest any visual similarity with any other birch species.
(3) ***Betula atrata*** a dark-barked diploid individual sampled in a tree line close to the village of Radostín (Českomoravská vrchovina), where Domin [27] described *B*. *atrata*. In the light of later findings [56], the individual should be classed as **atypical *B*. *pendula***. However, we needed to easily identify the position of this form of *Betula pendula* within the outcomes of the PCoA analysis, so we classed it separately.
(4) ***Betula pendula* with some morphological traits of *B*. *pubescens***: diploid individuals showing morphological features of *B*. *pendula* as well as those of *B*. *pubescens* (e.g. intermediate leaf shapes, reduced density of lenticels on branchlets, reduction in quantity or absence of pendulous branches, hairy branchlets or leaves). The birches classed in this way were sampled on sites suggesting the occurrence of *B*. *pubescens* (usually riparian stands, fringes of waterlogged sites and bogs at lower and middle elevations).
(5) ***Betula pendula* with some morphological traits of *B*. *carpatica***: diploid individuals showing morphological features of *B*. *pendula* as well as those of *B*. *carpatica* (e.g. intermediate leaf shapes, reduced density of lenticels on branchlets, reduction in quantity or absence of pendulous branches). Such individuals had to grow in a region and a habitat from which *B*. *carpatica* has been reported [26, 42].
(6) ***Betula oycoviensis*** diploid individuals from the population at the locality Volyně u Výsluní (S1 Fig). The sampled trees exhibited the morphological features of *B*. *oycoviensis*. They possessed a curved stem, dwarfed (“broomy”) crown and branches with a high abundance of epicormic shoots and dormant buds as well as (in contrast to *B*. *pendula*) markedly small leaves, see for example [57] for full details.
(7) ***Betula nana***: typical representatives of the species.
(8) ***Betula pubescens***: typical tetraploid representatives of the species.
(9) **Atypical *Betula pubescens***: tetraploid individuals showing most of the morphological traits indicative of *B*. *pubescens*, having, however, one or a few morphological features that are uncommon for the species (e.g. intermediate leaf shapes, semi-glabrous or glabrous branchlets). These atypical traits assessed together, nonetheless, did not suggest any marked visual similarity with any other birch species.
(10) ***Betula pubescens* with some morphological traits of *B*. *pendula***: tetraploid individuals showing morphological features of *B*. *pubescens* as well as those of *B*. *pendula*.
(11) **Tetraploids with intermediate morphological traits of *B*. *pubescens* and *B*. *carpatica***: tetraploid individuals showing morphological features of *B*. *pubescens* as well as those of *B*. *carpatica*. They were sampled in regions were both taxa are reported (overlapping species areas) and in habitats where both taxa could occur together (e.g. riparian stands, waterlogged sites and peat bogs of higher and mountain elevations).
(12) ***Betula carpatica***: tetraploid individuals, with morphological traits ascribed to the species in the literature [26, 58, 59] (e.g. glabrous abaxial face of leaf, double-toothed margin, ca 5 pairs of veins coming from midrib of leaf, more or less glabrous branchlets with sticky buds and reduced density of lenticels) that were sampled in regions, localities and habitats where the species has been recorded and documented in the literature (usually mountain sites). Trees classed as *B*. *carpatica* from research plots were also included (S2 Fig).
(13) ***Betula carpatica* with some morphological traits of *B*. *pendula***: tetraploid individuals showing morphological features of *B*. *carpatica* as well as those of *B*. *pendula*, occurring in regions from which both taxa are reported (overlapping species areas) and in habitats where both taxa could occur (e.g. mountain valleys).
(14) **Dark-barked tetraploids**: dark barked tetraploid individuals from the complex of birches closely related to *B*. *pubescens*, in the field mostly tentatively classed as dark-barked *B*. *carpatica*.
(15) **Tetraploids with attributes of *B*. *petraea***: tetraploid individuals sampled in the regions and habitats (stone fields and rock formations situated at middle elevations) where *B*. *petraea* has been recorded [26]. Such individuals showed at least some morphological traits ascribed to *B*. *petraea* (e.g. double-toothed leaves, patchily short velvet hairs close to blade veins, ca 8 pairs of veins coming from midrib of leaf). For a more detailed description see Sýkora [26]. Under this working unit we also included closer unspecified tetraploid birches sampled in rock towns of the Broumovsko region directly in places and locations where individuals of *B*. *petraea* had been previously recorded by Sýkora & Hadač [54]. The locations were chosen and sampled in collaboration with a local botanist.
(16) **Tetraploids showing some morphological traits of *B*. *nana***: tetraploid individuals whose morphology indicated some transition to traits of *Betula nana*. In fact, such individuals are ranked here because we suspected them of being triploids based on morphology; however, they were proven to be tetraploids by flow cytometry. Such individuals were recorded at some locations where tetraploid birches occur together with *B*. *nana* (Šumava, Jezerní slať and Horská Kvilda).
(17) ***Betula* ×*seideliana* (*B*. *carpatica* × *B*. *nana*)**: a triploid from the Šumava region (Jezerní slať) sampled, determined and provided to us by the local specialist Alois Pavličko (S3 Fig). Its identification was probably based on traits described in the primary source [60]. Nonetheless, our results suggest that *Betula* ×*seideliana* is in fact synonymous with *B*. ×*intermedia* (Hartm.) E. Thomas ex Gaudin, see below.
(18) **Unspeciefied triploids**: triploid individuals recorded and sampled in the Šumava Mts. (Paštěcké skály), the Třeboň region (Červené blato) and the Krušné hory Mts. (Volyně).

**Fig 3:**
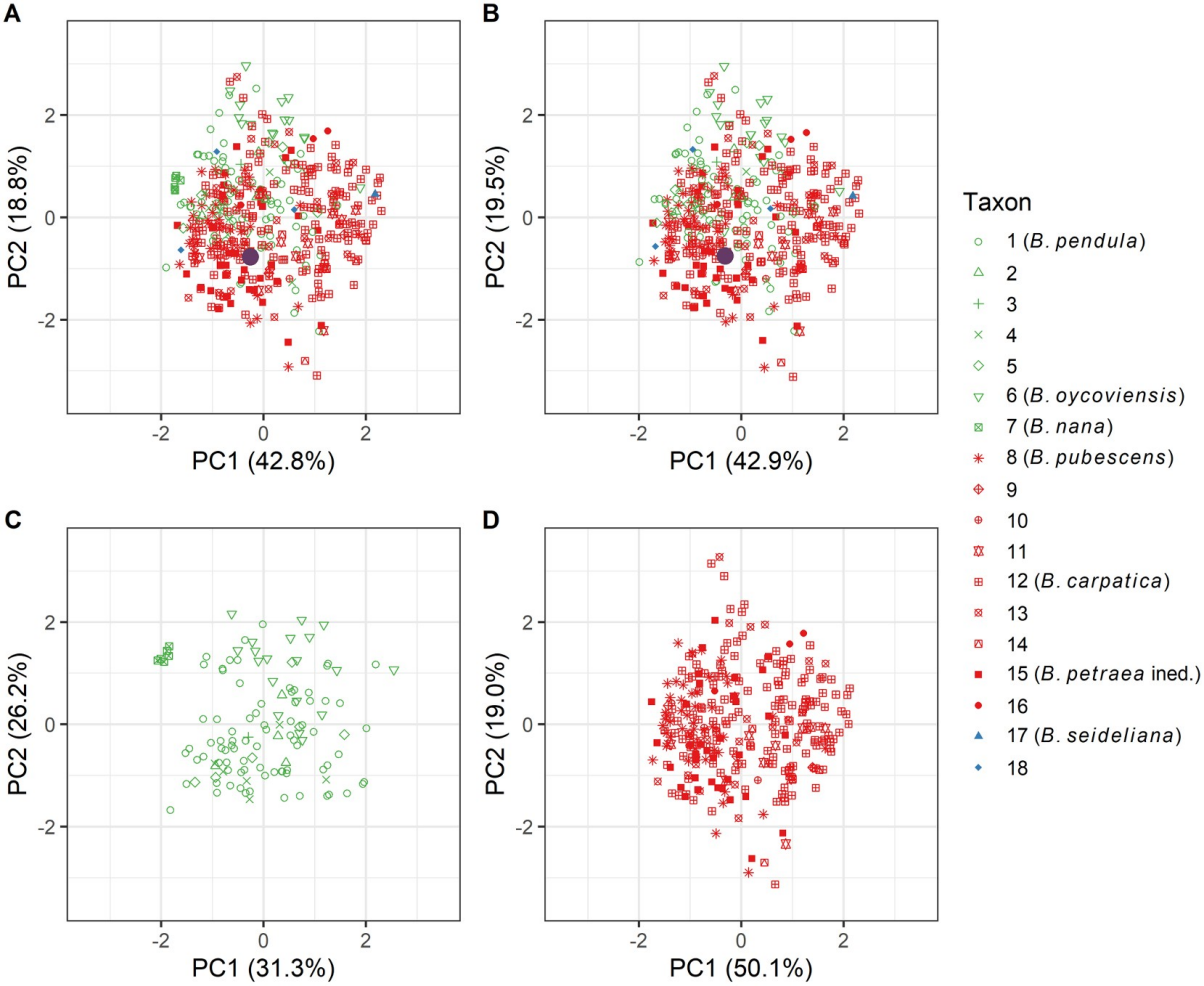
Principal component analysis (PCA) of morphological traits of sampled birch individuals. Subplots: A—all samples, B—all samples without B. nana, C—only diploid taxa, D—only tetraploid taxa. Diploid taxa and working units (1–7) are in green, tetraploids (8–16) are in red, and triploids (17 and 18) are in blue. The violet dots in plots A, B and D depict the position of the sample from Willdenow’s herbarium, on the basis of which *B*. *carpatica* was most probably described as a species, see the Discussion. The individuals included in the analysis were classified as described in the chapter Distinguishing and classification of sampled birches and the numbers in the legend correspond to the numbers of taxa and working units defined in the above-mentioned chapter.

The above classification must be viewed as an exclusively technical tool that was used for the purpose of a subsequent genetic evaluation of the sampled trees through molecular analyses. For instance, it has repeatedly been proven to extremely difficult or impossible to reliably distinguish tetraploid birches (*B*. *petraea*, *B*. *pubescens* and *B*. *carpatica*) from each other using the morphological traits described in the literature [61, 62], see also Fig 3. We, however, needed this classification to compare the genetic information on trees possessing morphological attributes of taxa such as *B*. *carpatica*, *B*. *petraea*, *B*. *oycoviensis* and *B*. *atrata* with genetic information of morphologically typical *B*. *pendula*, *B*. *pubescens* and *B*. *nana*.

### DNA sampling and obtaining microsatellite lengths

Collected samples were marked with unique IDs, placed in a cooling box and promptly transported to the laboratory, where they were stored in a deep freezer (-80 °C).

Genomic DNA was obtained from leaves collected across selected birch sites (Fig 2). Plant material (frozen in liquid nitrogen) was grinded by an oscillation mill (Retsch MM400). DNA was extracted using DNEasy Plant Mini Kit (QIAGEN) following the manufacturer’s protocol. The quantity and quality of DNA was evaluated spectrophotometrically and also checked on a 0.7% agarose gel. DNA samples were stored at -80 °C. For the subsequent PCR, the samples were diluted to 10 ng/μl.

Out of 50 tested loci taken from the studies by Kulju et al. [63], Tsuda et al. [64] and Tsuda et al. [65], 12 loci showed to be polymorphic and were chosen for whole analyses (S2 Table). Forward primers were fluorescence-labelled (NED, PET, VIC, 6-FAM) and optimized in two multiplex groups. The PCR reactions were run according to the relevant literature (see below).

The PCR reaction were run in 20 μl containing 15 ng of DNA, a set of primers (0.25 μM of each primer), 200 μM of dNTP, 2.5 mM MgCl_2_ and 1× buffer PCR multiplex mix with polymerase (Life Technologies, more recently ThermoFisher Scientific).

The PCR program [63] was set to initial 5 minutes of denaturation (95 °C), which activated the HotStar Taq polymerase, followed by 30 cycles of denaturation (95 °C, 60 s), annealing (57 °C, 75 s) and elongation (72 °C, 150 s). The final elongation step took 10 minutes (72 °C).

In the PCR program [64], 30 cumulative cycles were performed: denaturation (95 °C, 30 s), annealing (55 °C, 30 s) and elongation (72 °C, 45 s) with a final extension step (7 minutes, 72 °C).

We added 1 μl of the PCR product to the volume of 14 μl of a mixed solution of formamide with DNA ladder GeneTrace500 (Carolina Biolabs), prepared according to the manufacturer’s protocol. The solution was denatured (5 minutes, 95 °C) and quickly cooled on ice.

For determining SSRs lengths at each locus, a Genetic Analyser 3500 (Applied Biosystems, Foster City, California, USA) genetic sequencer was used. Raw sequence data were analysed with GeneMapper 4.1 (Applied Biosystems, Foster City, California, USA) and alleles were scored manually at each locus to ensure proper allele binning. For each sample at each locus, we identified the number of alleles (taking locus-specific stutter band and +A patterns into account). One allele represented homozygotes and more than one allele represented heterozygotes. Because the real allele dosage in tetraploids was not possible to infer unambiguously for most of the loci we prepared the matrix with observed alleles together with missing data up to four (except for the cases where only one allele was observed—these cases were treated as homozygotes with the allele dosage four).

### Data analysis

Similarities in the morphology of leaves were assessed by principal component analysis (PCA). The differences in genome size were tested by a Kruskal-Wallis test with subsequent multiple comparisons. The significance level α = 0.05 was chosen for all statistical tests.

Microsatellite data (separated by ploidy) were analysed with STRUCTURE version 2.3 [66–69] with the following settings: 20 repetitions for each K; 100,000 steps as burn-in followed by additional 100,000 Monte Carlo Markov Chain steps. An admixture model with correlated allelic frequencies was used as suggested in Porras-Hurtado et al. [70]. Other parameters were left at their default settings. For diploids, the analyses for K = 1–10 (one to number of groups in data + 3) were performed. Cluster analysis was not performed for triploid individuals due to the low number (4 pcs) of identified samples. For tetraploids, we ran analyses for K in the range from 1 to 12 (again, one to the number of groups + 3), and then, the real number of groups in the data for both diploids and tetraploids was estimated using the method suggested by Evanno et al. [71] via Structure Harvester [72]. Cluster matching and permutation was performed using CLUMPP [73]. Plots were created with Distruct [74].

For the visualization of genetic distances between all sampled individuals, regardless of their ploidy, principal coordinates analysis (PCoA) was performed. The input distance matrix (genetic distances) was computed based on the method suggested by Bruvo et al. [75]. This method is designed for computation of genetic distances between individuals irrespective of ploidy level. Allele counts (and the proportion of shared alleles) were determined separately for diploid and tetraploid birches. All computations related to the PCoA analysis and allele counts were performed in R 3.4.2 software [76] with the “polysat” package version 1.7–4 [77].

## Results

### Morphological analyses

As is apparent from the plot in Fig 3, in the morphology of leaves there is no strict separation of diploid and tetraploid birches. *B*. *nana* and *B*. *oycoviensis* are the only species that form more or less distinct groups.

### Basic genome size estimated using flow cytometry

**Fig 4:**
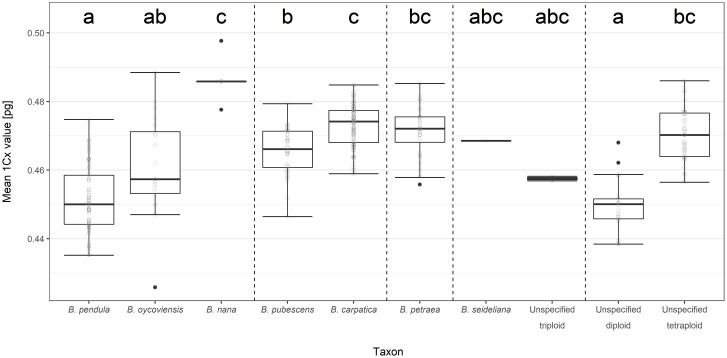
Distribution of 1Cx-values (x = 14) sensu Greilhuber et al. [53] calculated for birch taxa recognized in the Bohemian Massif. Unspecified triploids are descendants of unspecified different or single ancestral birch species. Unspecified diploids and tetraploids are mixtures of intermediate diploid (no. 2, 3, 4, 5) and tetraploid (no. 9, 10, 11, 13, 14, and 16) working units, respectively, as described in the chapter Distinguishing and classification of sampled birches of the Material and methods section. The box-and-whiskers plots are arranged according to Tukey’s standard design: Whiskers depict the minimum and maximum excluding outliers, black dots represent outliers (less than 1.5 times the lower quartile and more than 1.5 times the upper quartile, respectively). Empty dots stand for particular values in the dataset excluding outliers. Different letter indexes indicate significant differences at α = 0.05.

The distribution of 1Cx-values (x = 14) calculated for birch taxa of interest are summarized in Fig 4. Among diploids, the calculated mean 1Cx-values of *B*. *pendula* (0.451 pg) and *B*. *oycoviensis* (0.461 pg) were lower than that of *B*. *nana* (0.486 pg = 100%) by 7.2% and 5.1%, respectively. *Betula pendula* and *B*. *oycoviensis* significantly differed from *B*. *nana* in this parameter.

As for tetraploids, the calculated mean 1Cx-values of *B*. *pubescens* (0.466 pg) and *B*. *petraea* (0.471 pg) were lower than that of *B*. *carpatica* (0.473 pg = 100%) by 1.5% and 0.4%, respectively. The 1Cx-values of *B*. *pubescens* and *B*. *carpatica* were significantly different, although the difference was small. The 1Cx-value of *B*. *petraea* differed neither from *B*. *pubescens* nor from *B*. *carpatica*.

Out of the four triploids recorded within our study, one, classified as *B*. *seidelina* originating from Jezerní slať (Šumava Mts.), had a 1Cx-value (x = 14) of 0.469 pg, and the latter three from the Paštěcké skály (Šumava Mts.), Červené blato (Třeboň region) and Volyně u Výsluní (the Krušné hory Mts.) localities exhibited 1Cx-values (x = 14) of 0.458 pg, 0.457 pg and 0.459 pg, respectively.

### Molecular analyses

First, the proportion of alleles shared between diploids and tetraploids was computed to find out if the tetraploid taxa under study are of autopolyploid or allopolyploid origin. In total, we recorded 191 alleles out of which 128 (67%) alleles were shared between diploid and tetraploid taxa. Altogether 10 alleles were recorded exclusively in diploids and 53 alleles were found only in tetraploids (Table 2).

**Table 2:** The number of unique alleles found within diploid and tetraploid birches, the number of alleles shared between the two ploidy levels, and total number of alleles in the twelve microsatellite loci used to genotype 432 samples. For a detailed description of loci, see S2 Table.

Locus	Unique alleles		Shared alleles	Total allele count
	Diploids	Tetraploids		
L31	2	3	8	13
L54	0	2	18	20
L78	0	4	12	16
L022	1	4	16	21
L71	0	7	8	15
Bema7	1	3	6	10
L012	2	12	9	23
Bema1	0	3	3	6
Bema11	1	5	7	13
L27	0	5	20	25
Bepl3	2	2	10	14
L34	1	3	11	15
**Sum**	**10**	**53**	**128**	**191**

**Fig 5:**
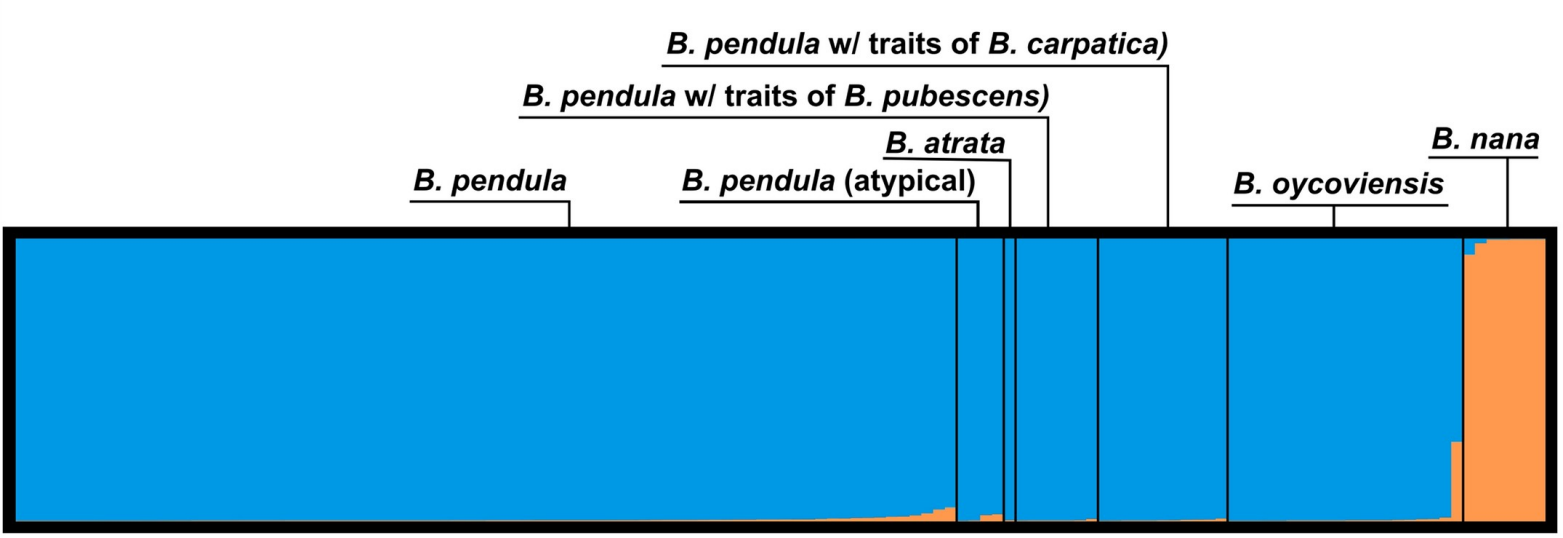
Result of clustering analysis in STRUCTURE software (K = 2) for diploid individuals of the genus Betula from the Bohemian Massif (Czech Republic). The Y axis shows the probability of affiliation to a certain group depicted by colour for every individual. Each individual is one bar (X axis). Two clusters were differentiated: (1) *B*. *pendula*, *B*. *oycoviensis* and related taxa, and (2) *B*. *nana*.

Estimation of the number of groups among diploid samples according to Evanno et al. [71] showed that two clusters is the most probable solution (see S3 Table). Clustering analysis of diploid individuals (software STRUCTURE) in Fig 5 showed a clear pattern for K = 2: (1) *B*. *pendula*, *B*. *oycoviensis* and related taxa, and (2) *B*. *nana*.

**Fig 6:**
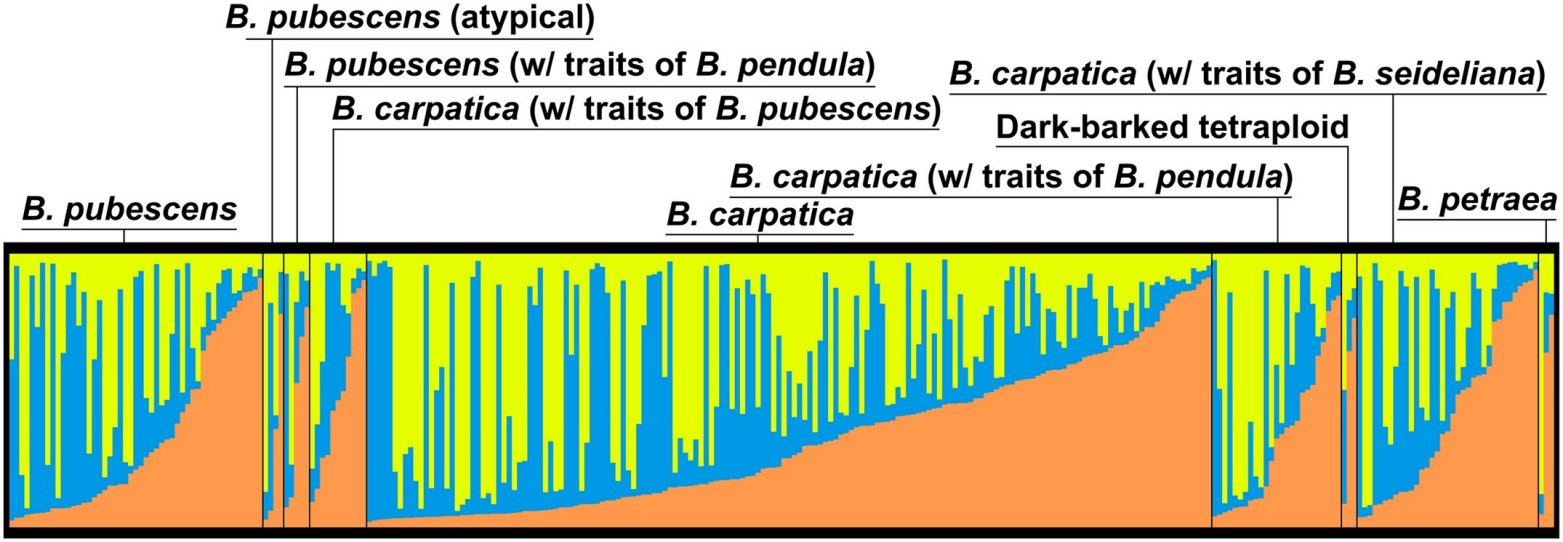
Result of clustering analysis in STRUCTURE software for tetraploid individuals of the genus *Betula* from the Bohemian Massif (Czech Republic). The Y axis shows the probability of affiliation to a certain group depicted by colour for every individual. Each individual is one bar (X axis). No pattern corresponding with species determination was observed; all tetraploid samples formed one compact cluster.

**Fig 7:**
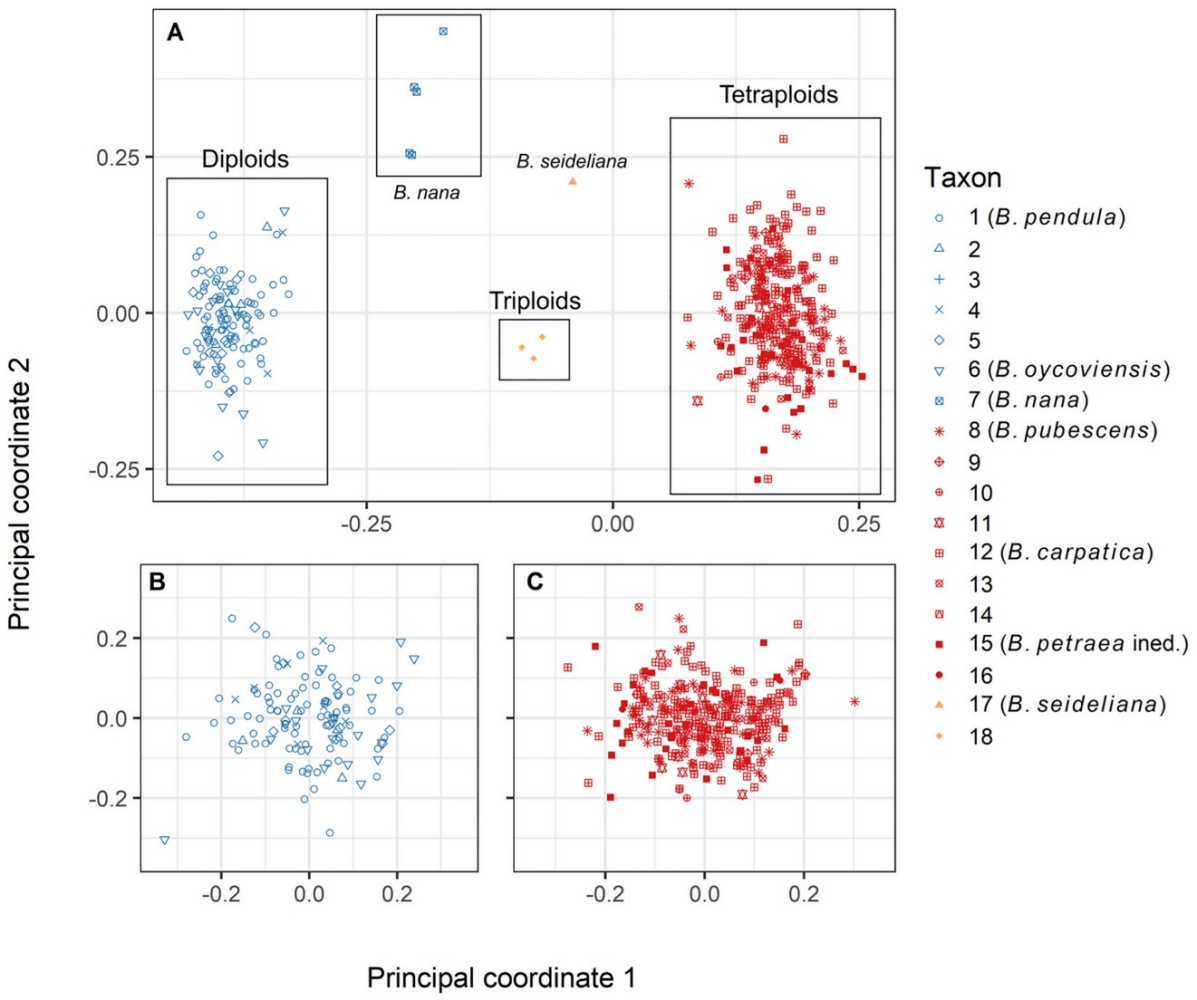
Principal coordinates analysis (PCoA) of genetic distances of the genus *Betula* in the Bohemian Massif (Czech Republic) for 130 sampled diploid (blue), 298 tetraploid (red) and 4 triploid (orange) individuals (A), only diploids without *B*. *nana* (B), and only tetraploids (C). The depicted individuals were classified as described in the chapter Distinguishing and classification of sampled birches and the numbers in the legend correspond to the numbers of taxa and working units defined in the chapter.

In the case of tetraploids, the first applied analysis according to Evanno et al. [71] estimated that there were most probably three groups in the dataset, see S4 Table. However, the results simultaneously showed a mixture of group assignments for all taxa and “working units” which suggests that all samples technically belongs to one homogenous group with no real structure. The hypothesis of one homogenous tetraploid group cannot be tested using the Evanno method, because it is not possible to compute the difference of mean P(K). Subsequent clustering analysis did not show any pattern related to the determination of the analysed taxa and “working units” (Fig 6). In the end, the PCoA analyses revealed that only one homogenous group of tetraploids is the most probable scenario (Fig 7).

Among diploids, *B*. *pendula* **(1)**, the “atypical” form of *B*. *pendula* **(2)**, *B*. *atrata* Domin **(3)**, *B*. *pendula* with some morphological traits of *B*. *pubescens* **(4)**, *B*. *pendula* with some morphological traits of *B*. *carpatica* **(5)** and *B*. *oycoviensis* **(6)** formed a homogenous cluster in space projected by PCoA analysis (blue clusters in the plots in Fig 7). There is no visible pattern corresponding with working unit assignment among these diploid taxa. The absence of any pattern in assignment of minor diploid taxa closely related to *B*. *pendula* (no. 1–6) was confirmed also if the PCoA was conducted exclusively for them, that is, without *B*. *nana* and tetraploids (Fig 7, plot B).

*Betula nana* **(7)** proved to be clearly distinctive from the other diploid taxa and rather closer to the cluster of tetraploids on the right side of the PCoA plot than the diploid birches belonging to the *B*. *pendula* complex (Fig 7, plot A).

Tetraploid *B*. *pubescens* **(8)**, the “atypical” form of *B*. *pubescens* **(9)**, *B*. *carpatica* **(12)**, *B*. *carpatica* with some morphological traits of *B*. *pendula* **(13)** and tetraploids showing some morphological traits of *B*. *nana* **(16)** were not distinguishable from each other in the analysis (Fig 7, plots A and C). *Betula pubescens* with morphological traits of *B*. *pendula* **(10)** and tetraploids with mixed traits of *B*. *carpatica* and *B*. *pubescens* **(11)** did not form a separate cluster, although some of the individuals included as these working units were on the margin of the main tetraploid cluster. Unidentified dark-barked tetraploid **(14)** appeared to be approximately in the middle of the tetraploid cluster. Tetraploids with attributes of *B*. *petraea* **(15)** and tetraploids showing some morphological traits of *B*. *nana* **(16)** appeared within the tetraploid cluster as well.

To summarize, we were unable to distinguish more groups of birches within the tetraploid cluster (*B*. *pubescens* complex). The absence of any pattern in the assignment of tetraploid taxa closely related to *B*. *pubescens* (**8–16**) was confirmed also if the PCoA was conducted exclusively for tetraploids, that is, without *B*. *nana* and diploids.

*B*. *×seideliana* **(17)** appeared between the cluster of tetraploids and the cluster of *B*. *nana*, as expected, since this taxon is described as their triploid hybrid. Also the unspecified triploids **(18)** from the localities Volyně u Výsluní (Krušné hory Mts.), Paštěcké skály (Šumava Mts.) and Červené Blato (Třeboň region) appeared between the diploid and tetraploid clusters. The unspecified triploids were, however, clearly differentiated from *B*. *×seideliana*, which suggests that these plants were crossbreeds of individuals belonging to the *B*. *pendula* complex (main diploid cluster) and *B*. *pubescens* complex (tetraploid cloud), see Fig 7, plot A.

## Discussion

We intended to assess the position of *B*. *carpatica* in the context of *B*. *pubescens* and some “minor” birch taxa reported from the Bohemian Massif.

### Morphological analyses

Our simplified morphological analysis indicates that it can be difficult to determine the ploidy level of birches in the field, let alone to identify minor taxa (see Fig 3). This is in agreement with statements, for example, by Hynynen et al. [78] and Anamthawat-Jónsson and Thórsson [13]. As mentioned in the Material and Methods, the limitations of the approach applied in our study stem from the necessity to include only such foliar traits that can be measured for all birch taxa considered here. Some more detailed and complex morphological studies have suggested relatively reliable approaches to determining ploidy levels using morphology [49, 61, 62]. The level of reliability in distinguishing the diploids and tetraploids using morphometry ranged from 74 to nearly 100% depending on the method and region. Neither of the above-mentioned morphometric studies, however, was able to identify minor tetraploid taxa.

### Basic genome size estimated using flow cytometry

At the level of basic genome size 1Cx (x = 14), the differences between the birch taxa under assessment are not big. For example, the relative difference in mean 1Cx-values between diploid *B*. *pendula* and *B*. *nana* is 7.2%. This is less than what was recorded between populations of other plant species across larger geographic ranges [79, 80]. Although the relative difference in basic genome size between *B*. *pendula* and *B nana*, both of which are broadly accepted species, is relatively small, it can serve as a benchmark for evaluating differences between other species. Between tetraploid *B*. *pubescens* and *B*. *petraea*, the difference in mean 1Cx-values is insignificant and 6.5-times smaller (1.1%) than that between *B*. *nana* and *B*. *pendula*. Between tetraploid *B*. *carpatica* and *B*. *pubescens*, the difference in mean 1Cx-values is 4.8-times smaller (1.5%), although the statistical test labelled it as significant.

The difference in 1Cx-values between *B*. *pubescens*, *B*. *petraea* and *B*. *carpatica* could be indicative of some degree of adaptation to a gradient in environmental conditions, as tetraploid birches from higher altitudes and colder climates tend to have slightly greater basic genome size. This has been described, for example, in pines [81]. On the other hand, it is not reasonable to advocate the concept of *B*. *carpatica* or *B*. *petraea* as species separate from *B*. *pubescens sensu lato* based solely on this small difference, especially considering the results of the molecular analyses. Moreover, this small difference could also easily be a result of some level of methodological inaccuracy.

The proportion of triploids recorded in our research (ca 1%) was significantly lower than that reported, for example, in Iceland [13, 82], where *B*. *pubescens* crosses with *B*. *nana*. One possible explanation is the fact that populations of *B*. *nana* in the Bohemian Massif are very small and isolated. Our results, however, indicate a possibility of hybridization also between diploid *B*. *pendula* and tetraploid taxa, albeit very rare. Regarding this, see Figs 4 and 7, plots A and C, where the triploids from the localities Volyně u Výsluní, Paštěcké skály and Červené blato are significantly apart from *B*. *×seideliana* and more distant from *B*. *nana*.

The very low percentage of triploids recorded in our research stands in contrast to the high morphological variability and “intermediality” of the analysed birches. Many of them exhibited certain morphological traits between the tetraploid *B*. *pubescens* complex and diploid *B*. *pendula* (or patchily *B*. *nana*). Interestingly, such morphologically intermediate individuals were mostly not triploid, because, as mentioned above, we recorded only four triploid birches out of 432. Our findings in this regard correspond to the observations of Gill & Davy [83].

Ashburner & McAllister [9] suggest that *B*. *pendula* could contribute genes to the gene pool of the tetraploid *B*. *pubescens* through the production of unreduced gametes by the diploid and diploid and triploid gametes by triploid hybrids. How much this hypothesis could explain the observations described in the previous paragraph will have to be answered by future research.

### Molecular analyses and their outcomes in the light of literature review

The molecular methods used were not able to identify significant differences between *B*. *pubescens*, *B*. *carpatica* and *B*. *petraea* (Figs 6 and 7, plots A and C). In light of our molecular results, *B*. *oycoviensis* seems to belong to the *B*. *pendula* complex (Figs 5 and 7, plots A and B), which is in congruence with the results of flow cytometry but contradicts its more or less distinctive morphology described, for example, by Balas et al. [57]. The results of our molecular analyses support the opinion of Ashburner & McAllister [9] that *B*. *oycoviensis* is of low taxonomic value. Among diploids, *B*. *nana* is naturally clearly distinct in terms of morphology and genome size, and it also differs from the other birches in molecular markers. The broadly accepted native species *B*. *nana* could therefore serve as an example of completed speciation and as a benchmark for assessing the taxonomic importance of *B*. *carpatica* and other minor taxa in the diploid and tetraploid clusters. Interestingly, plot A in Fig 7 shows that individuals of the diploid species *B*. *nana* and rather closer to the cluster of tetraploids on the right side of the PCoA plot than the diploid birches belonging to the *B*. *pendula* complex. Molecular methods also indicate that individuals of *B*. *nana* sampled in the Jizerské hory Mts. are more distant from the cluster of diploids than *B*. *nana* sampled in the Šumava and Krušné hory Mts. (Fig 7, plot A), suggesting population variability.

### Review of relevant historical sources on *B*. *carpatica*

The lack of any distinct morphological or molecular traits of *B*. *carpatica* directed our attention to the history of this taxon. *Betula carpatica* as a species was probably suggested by Pál Kitaibel (presumably together with Franz de Paula Adam von Waldstein) and surely approved and introduced to the taxonomic system by Carl Ludwig Willdenow at the beginning of the 19th century. The taxon was attributed to the mentioned authors in the fourth supplemented edition of *Caroli Linné Species Plantarum* (p. 464) by Willdenow [84]. In this Willdenow’s edition (1805), there is the hint “pl. rar. hung.”(placed after the scientific name *Betula carpatica* Waldst. et Kitaib.) possibly referring to the *Descriptiones et icones plantarum rariorum Hungariae* [85, 86], where, nonetheless, *B*. *carpatica* is not mentioned. On the other hand, Waldstein and Kitaibel were in touch with Willdenow and consulted their herbarium specimens with him [87, 88].

**Fig 8:**
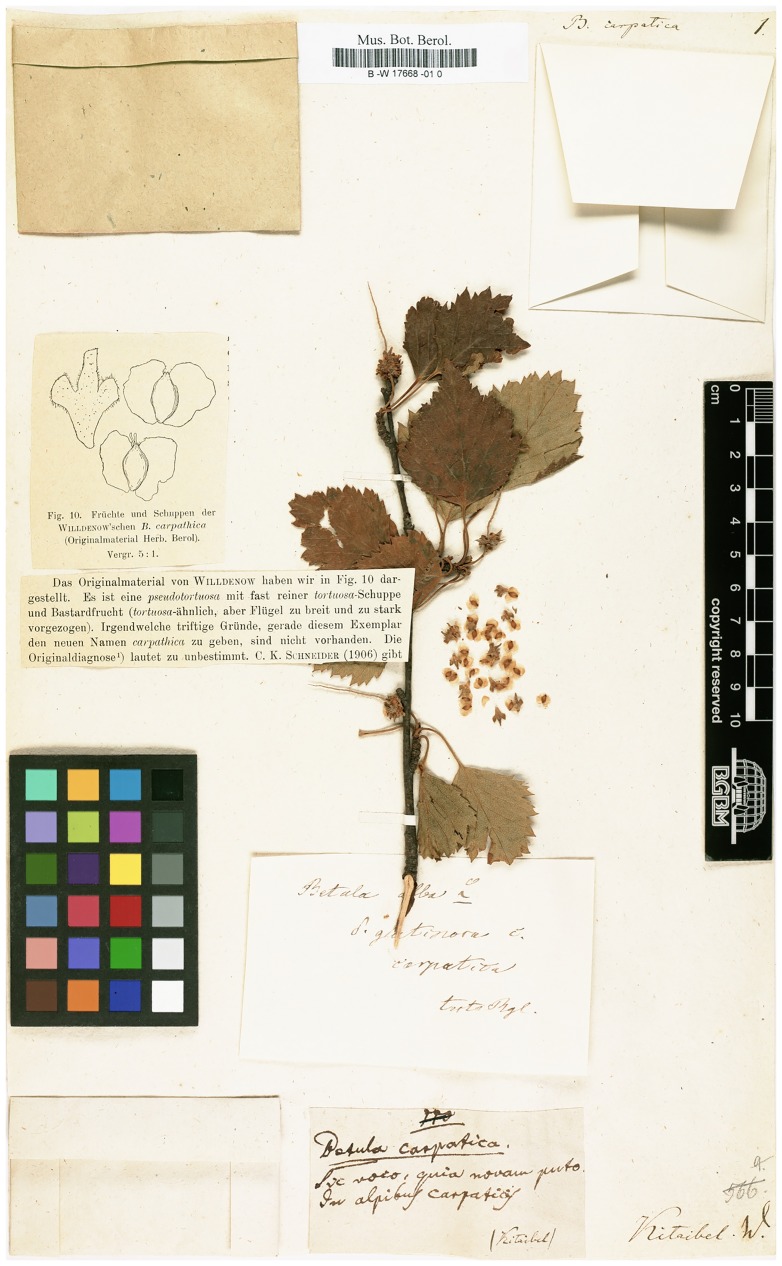
The only specimen of *Betula carpatica* in Willdenow’s collection. The hand-written note at the bottom was authored by Pál Kitaibel. The type-written comments and pen drawing of seeds and catkin scales were added to the specimen on the middle-left side of the figure originate from Morgenthaler’s [95] study. Source: Curators of Herbarium B (2017). Digital specimen images at the Herbarium Berolinense. [Dataset]. Version: 14 Aug 2017. Data Publisher: Botanic Garden and Botanical Museum Berlin. http://ww2.bgbm.org/herbarium/ [BW17668010, image ID: 398019.]

In Willdenow’s herbarium collection preserved in Berlin (now a part of collections of the Herbarium Berolinese der Freier Universität in Berlin), there is a specimen (Röpert 2000+ [continuously updated]) determined as *Betula carpatica* with a tag containing a hand-written note “*Betula carpatica*. ?*Hic*? *voto quia novam puto in alpibus Carpaticis*” (see Fig 8). We addressed the authorities from the herbarium collection management to learn more about the specimen (personal communication with Sarah Bollendorf and Paul Hiepko) and learned that the questioned item is the only specimen of *B*. *carpatica* in Willdenow’s collection and that the Latin note on the tag was written by Kitaibel (supposedly except for Kitaibel’s name in brackets). Therefore, we hypothesise that Kitaibel (and probably Waldstein) suggested *B*. *carpatica* through their herbarium specimen submitted to Willdenow, who subsequently classified or approved it as a new species, included it to his own herbarium and also his supplemented edition of *Species Plantarum*.

After its inclusion in Willdenow’s edition of *Species Plantarum*, Carpathian birch (*B*. *carpatica*) became generally accepted as a species by many floristic and taxonomic works originating in Central and Western Europe [89–92].

Nonetheless, already during the 1800s some botanists questioned Willdenow’s taxonomic/systematic ranking of *B*. *carpatica*. Friedrich Wimmer in his report [93] considered *B*. *carpatica* a mere form of *B*. *pubescens* resulting from the harsh mountain environment. Besides Wimmer, Willkomm [94], too, regarded *B*. *carpatica* as a product of a mountain climate.

In 1915, Morgenthaler [95] concluded that it was no longer justifiable to use the “old broad” term *B*. *carpatica*. For instance, he classed all individuals in the Swiss Alps previously considered *B*. *carpatica* as pure *B*. *pubescens*. Within his study, he also inspected the original Willdenow’s specimen of *B*. *carpatica* at the Herbarium Berolinense (Fig 8) and stated that there were no satisfactory reasons to give Willdenow’s specimen the specific name *B*. *carpatica*.

The traits for the determination of *B*. *carpatica* mentioned in the literature are inconclusively defined. It is worth recalling that it was the very specimen in Willdenow’s herbarium on the basis of which *B*. *carpatica* was most probably ranked as a species; that is, the holotype sensu McNeill [96]. As Steenis [97] noted, deductions derived from herbarium material (which were most probably made in the case of *B*. *carpatica*) must of necessity be limited.

### Overall synthesis

If we combine all aforesaid pieces of information with our molecular results (Fig 7) for material sampled in the Czech Republic, the treatment of *B*. *carpatica* as a species was highly disputable. Similarly, Ashburner & McAllister [9] note that populations of *B*. *pubescens* growing in the Carpathians named *Betula carpatica* do not differ significantly from *Betula pubescens* var. *pubescens*.

An analogous problem surrounds the determination of *B*. *petraea* sensu Sýkora [26] (Fig 7). The question of the possibility to distinguish *B*. *petraea* from *B*. *pubescens* is raised also by Skokanová [18]. She inspected a population of tetraploid birch occurring by the Jurské jezero lake in the Malé Karpaty Mts. (Slovakia) that was previously classified by Sýkora [26] as typical *B*. *petraea*. Skokanová came to the conclusion that the tetraploid individuals from the Jurské jezero lake are representatives of pure *B*. *pubescens*, or *B*. *pubescens* subsp. *pubescens* sensu Olšavská [98].

The latest treatment of genus *Betula* in the updated Key to the flora of the Czech Republic [99] changes the ranking of *B*. *carpatica* from specific to subspecific level [28]. The proposed treatment also keeps omitting *B*. *petraea* from the list of accepted birch taxa and calls for further research. Our results in fact support the shift in view on the tetraploid birches proposed in the key. Even we suggest distinction of *B*. *carpatica* on lower taxonomic level than subspecies. In general, all the tetraploid birches sampled within our study, including *Betula pubescens*, *B*. *carpatica*, and *B*. *petraea* and also all the morphologically intermediate and dark-barked tetraploid forms (working units), created one cloud in the PCoA diagram (Fig 7) that is clearly distinguished from diploids. The reliable recognition of minor tetraploid taxa and “working units” from each other was possible neither by morphological means [61, 62] Fig 3 nor by the application of molecular genetics (Figs 6 and 7).

Using the hierarchy of species, subspecies, varieties and forms aptly outlined by Steenis [97], we would rank *B*. *carpatica* and *B*. *petraea* from the Bohemian Massif somewhere between a variety and a form of *B*. *pubescens*. Possibly these two taxa are closer to being forms because they are merely “somehow” different from “pure” *B*. *pubescens*, but it is difficult to define reliable morphological traits usable for unambiguously distinguishing these forms from *B*. *pubescens* and from each other.

It is important to reiterate at this point that the individuals were (tentatively) classified as *B*. *carpatica* and *B*. *petraea* sensu Sýkora (and thus distinguished from *B*. *pubescens*), if they showed several traits ascribed to these taxa in the literature [26, 42, 43, 59] and simultaneously if they grew at the localities from which *B*. *carpatica* and *B*. *petraea* sensu Sýkora were reported. At such localities of reported occurrence, attention was given to tetraploids. Therefore, if some really pure and typical *B*. *carpatica* and *B*. *petraea* sensu Sýkora existed, they were with great probability sampled and included in our analyses, at least in several cases.

The results of the present study document that the diploid species *B*. *pendula* and *B*. *nana* can be easily genetically distinguished from each other (Figs 5 and 7). Interestingly, *B*. *nana* appeared to be closer to the cluster of tetraploids on the right side of the PCoA diagram than *B*. *pendula*. This could explain why hybridization and backcrossing as well as the presence of triploids is more frequent, for example, in Iceland, where *B*. *nana* is common [13, 100]. Another possible explanation could reside in direct gene flow from the diploid to the tetraploid level through the fertilization of *B*. *pubescens* female gametes by unreduced *B*. *nana* pollen [11].

Our molecular results suggest that individuals of *B*. *oycoviensis* Besser from the Volyně u Výsluní locality (Krušné hory Mts.) and *B*. *atrata* Domin from Radostín (Českomoravská vrchovina highland) do not differ from *B*. *pendula* (Figs 5 and 7) despite being significantly distinguishable using morphology. Our observation supports the conclusion of Hejtmánek [56] refuting the classification of *B*. *atrata* Domin as a species. Regarding *B*. *oycoviensis* Besser, our outcome corresponds with the opinions of Ashburner & McAllister [9] and newly also [28], who consider *Ojców birch* as a weak-growing and heavily fruiting form of *B*. *pendula*. A more detailed study with more individuals, including ones from Polish populations is nonetheless required, as only a few individuals naturally occurring in the Bohemian Massif were analysed in our study.

The overall plasticity of molecular data in the tetraploid complex of *B*. *pubescens* and diploid complex of *B*. *pendula* is explainable by the continuity of birch occurrence in the area, including glacial periods [101–103] as well as by easy seed dispersal (anemochory) of potent birches. Therefore, the formation of isolated endemic populations is improbable. On the other hand, the specificity of the environment in some habitats and continuous natural selection under extreme conditions at some sites could play a significant role in the development of some birch forms or ecotypes, and this should be borne in mind, for example, when acquiring reproduction material for forestry and natural protection purposes.

Since the invention of binomial nomenclature by Linné, there has been a conflict between “splitters”, who name more or less well defined local populations as separate species, and “lumpers”, who give less attention to geographic variation and unite local variants into single species [104]. In other words, the species problem is often much more a problem of species delimitation than of species definition. The species problem may not be primarily a theoretical issue but a problem of taxonomic practice [105].

Because of high morphological variability, Morgenthaler [95] needed many measurements of various morphological traits to classify birch specimens to the “taxonomic groups” into which he dissected the continual morphologic spectrum between “pure” *B*. *pendula* and “pure” *B*. *pubescens*, which are clearly genetically distinct species differing also in ploidy level. Therefore, the question remains whether it is even theoretically possible to reliably identify *B*. *carpatica* and *B*. *petraea* by morphological means, given that we were unable to separate these species from *B*. *pubescens* using molecular methods.

The greatest problem concerning *B*. *carpatica* is that it has been delimited as a species exclusively on the basis of morphological traits probably of a single specimen in 1805, that is, in the essentialist era before Darwin and Mendel. In our opinion, it is more a “traditional”, invalid species than an actual taxon. From the viewpoint of conservation management and diversity protection, this does not mean that mountain populations of *B*. *pubescens* up to now classified as *B*. *carpatica* should be less worthy of protection because they are no longer regarded as a local minor species. On the contrary, we should learn to be aware of and value biodiversity at all levels of the hierarchy of life, even in legislation [104]. This is surely a better approach than (either because of conservation or because of tradition) to rank local forms as species. In forestry, too, it seems much more important to distinguish between *B*. *pendula*, *B*. *pubescens* and *B*. *nana* and respect local birch populations and ecotypes than to try and recognize “minor” taxa that are often unrecognizable. Respecting local provenances within broadly conceived species is in our opinion more beneficial focusing on hard-to-distinguish minor taxa.

## Conclusions

We were not able to distinguish any taxonomic group within the tetraploid cluster of birch species (the *B*. *pubescens* complex) in the Bohemian Massif. The molecular methods used in our study did not detect any significant differences between *B*. *carpatica*, *B*. *petraea* and *B*. *pubescens*. Basic genome size estimated using flow cytometry did not allow us to reliably distinguish any tetraploid taxon.

Our results corroborate the conclusions of previous studies searching for morphological traits of purportedly different tetraploid birches [61, 62]. Based on these results, a literature review and a study of herbarium specimens, we conclude that *B*. *carpatica* and *B*. *petraea* should not be classified as distinct at the species level at least in the surveyed area. Using the hierarchy of species, subspecies, varieties and forms delineated by Steenis [97], we would rank *B*. *carpatica* and *B*. *petraea* in the Bohemian Massif somewhere between varieties and forms of *B*. *pubescens*, probably closer to the latter. Tetraploid birches occurring in the Bohemian Massif are most probably allopolyploid. This is evidenced by the presence of unique alleles in tetraploid birches that are absent in diploids. The allopolyploidy of these tetraploid birches is indicated also by a stable difference in holoploid genome size (1Cx value) between diploids and tetraploids.

Within the *B*. *pendula* complex, no taxonomic group can be differentiated with certainty. Results of molecular methods indicate that individuals of *B*. *oycoviensis* from the locality Volyně u Výsluní (Krušné hory Mts.) do not differ from *B*. *pendula* to an extent that would merit their classification as a species. In our opinion, their separation is not justified, even though *B*. *oycoviensis* is distinguishable morphologically. However, more research into populations assigned to this taxon across its entire distribution range is necessary to reach a definite conclusion on its taxonomic position. The complex of minor diploid taxa closely related to *B*. *pendula* and the cluster of tetraploids closely related to *B*. *pubescens* can be easily distinguished from the broadly accepted native species *B*. *nana* based on molecular analyses as well as in terms of morphology.

We recorded a very low percentage of triploids, which stands in contrast to the vast morphological variability and “intermediality” of the analysed birches. However, our study indicates that both *B*. *nana* and *B*. *pendula* are able to form triploid hybrids with tetraploid birches in the Bohemian Massif, albeit very rarely.

## Supporting information

S1 Fig Betula oycoviensis. Upper left and upper right: Individuals from the locality Volyně u Výsluní. Photos by Baláš (2013). Lower left: Detail of leaves of *Betula oycoviensis*. Photo by Baláš (2018). Lower right: An individual in the Chomutov ZOO (Northern Bohemia). The individual was transplanted to Chomutov from Volyně u Výsluní in 1996. Photo by Kuneš (2018).
(PDF)

S2 Fig Betula carpatica. Upper left: an individual at the bottom of a glacial carved valley in the Krkonoše Mts. Photo by Kuneš (2010). Upper right: an individual at an avalanche slope of a corrie in the Krkonoše Mts. Photo by Kuneš (2010). Lower left: an individual on a mountain peat bog in the Jizerské hory Mts. Photo by Baláš (2010). Lower right: an individual in the experimental plantation in the Jizerské hory Mts. Photo by Kuneš (2018).
(PDF)

S3 Fig *Betula ×seideliana* sensu Missbach 1908. Photos taken by Pavličko (2012) in the Šumava Mts.
(PDF)

S1 Table The list of sampled localities including general characteristics, classification of habitats sensu Chytrý et al. [47] as well as forest site classification sensu Viewegh et al. [48]. (XLSX)

S2 Table Description of used microsatellite markers. (XLSX)

S3 Table Estimation of the number of groups present among diploid samples. The method as well as the meanings of the symbols used in the table are described by Evanno et al. [71]. The most probable scenario is highlighted in grey.
(XLSX)

S4 Table Estimation of the number of groups present in the tetraploid *Betula* dataset according to the method proposed by Evanno et al. [71]. Most probable cases are highlighted in grey; however, the hypothesis of one homogenous tetraploid group technically cannot be tested using this method.
(XLSX)

S1 File Genome_size_data.xlsx. Genome size (obtained by flow cytometry) dataset.
(XLSX)

S2 File Data_morphometrics.csv. Measured morphometric parameters on study individuals.
(CSV)

S3 File Diploids.txt. Allele calls for diploid individuals.
(TXT)

S4 File Triploids.txt. Allele calls for triploid individuals.
(TXT)

S5 File Tetraploids.txt. Allele calls for tetraploid individuals.
(TXT)
