# Supplementary material for: Is Betula carpatica genetically distinctive? A morphometric, cytometric and molecular study of birches in the Bohemian Massif with a focus on Carpathian birch
Source: PLoS One. 2019 Oct 31;14(10):e0224387. doi: 10.1371/journal.pone.0224387 (PMC6822711; doi:10.1371/journal.pone.0224387)
Supplement: S1 Fig — Upper left and upper right: Individuals from the locality Volyně u Výsluní. Photos by Baláš (2013). Lower left: Detail of leaves of Betula oycoviensis. Photo by Baláš (2018). Lower right: An individual in the Chomutov ZOO (Northern Bohemia). The individual was transplanted to Chomutov from Volyně u Výsluní in 1996. Photo by Kuneš (2018). (PDF) [file pone.0224387.s001.pdf]

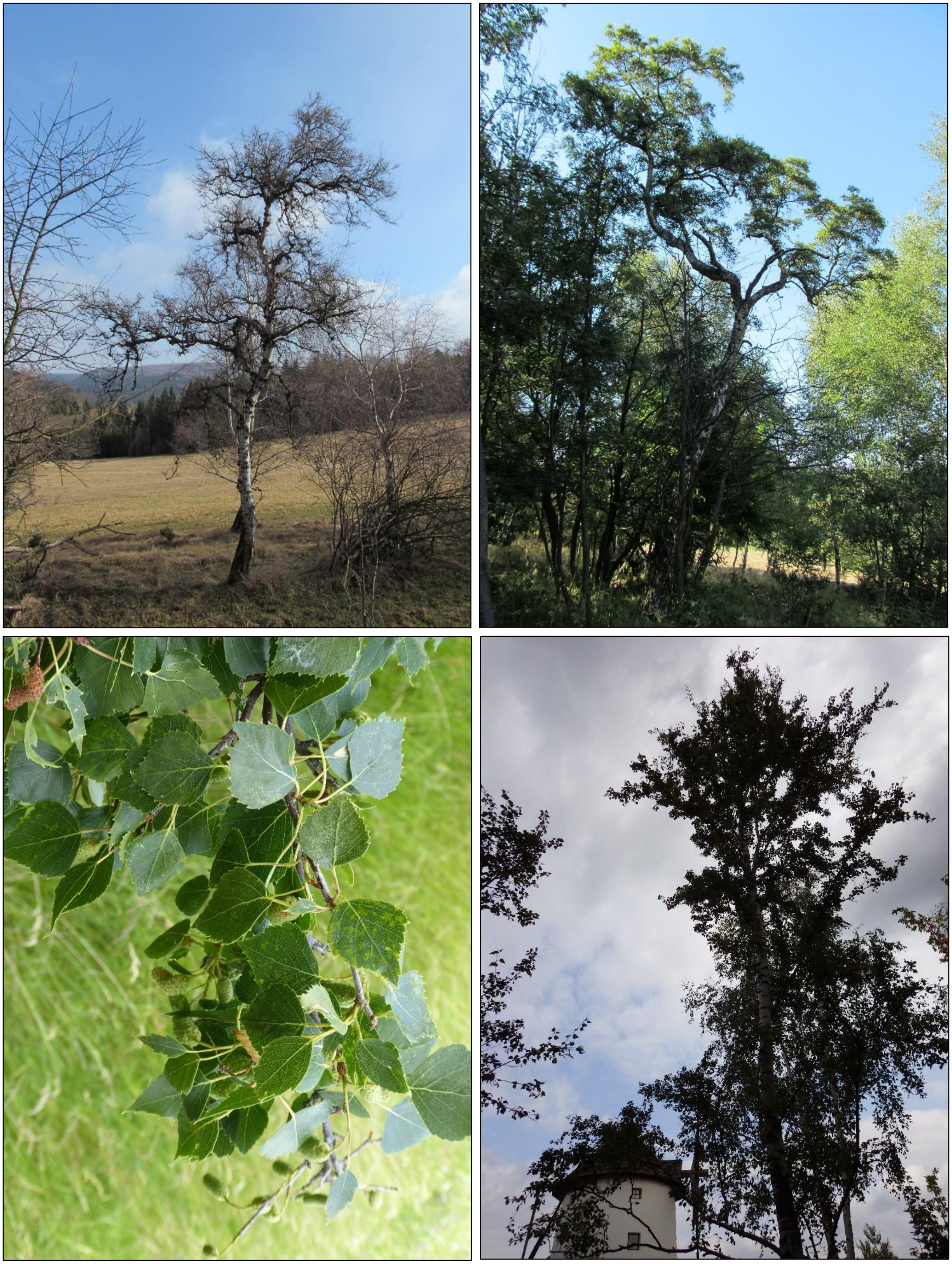

S1 Fig. *Betula oycoviensis.*
Upper left and upper right: Individuals from the locality Volyně u Výsluní. Photos by Baláš (2013).
Lower left: Detail of leaves of *Betula oycoviensis*. Photo by Baláš (2018). Lower right: An individual in the Chomutov ZOO (Northern Bohemia). The individual was transplanted to Chomutov from Volyně u Výsluní in 1996. Photo by Kuneš (2018).
