# Supplementary material for: Is Betula carpatica genetically distinctive? A morphometric, cytometric and molecular study of birches in the Bohemian Massif with a focus on Carpathian birch
Source: PLoS One. 2019 Oct 31;14(10):e0224387. doi: 10.1371/journal.pone.0224387 (PMC6822711; doi:10.1371/journal.pone.0224387)
Supplement: S2 Fig — Upper left: an individual at the bottom of a glacial carved valley in the Krkonoše Mts. Photo by Kuneš (2010). Upper right: an individual at an avalanche slope of a corrie in the Krkonoše Mts. Photo by Kuneš (2010). Lower left: an individual on a mountain peat bog in the Jizerské hory Mts. Photo by Baláš (2010). Lower right: an individual in the experimental plantation in the Jizerské hory Mts. Photo by Kuneš (2018). (PDF) [file pone.0224387.s002.pdf]

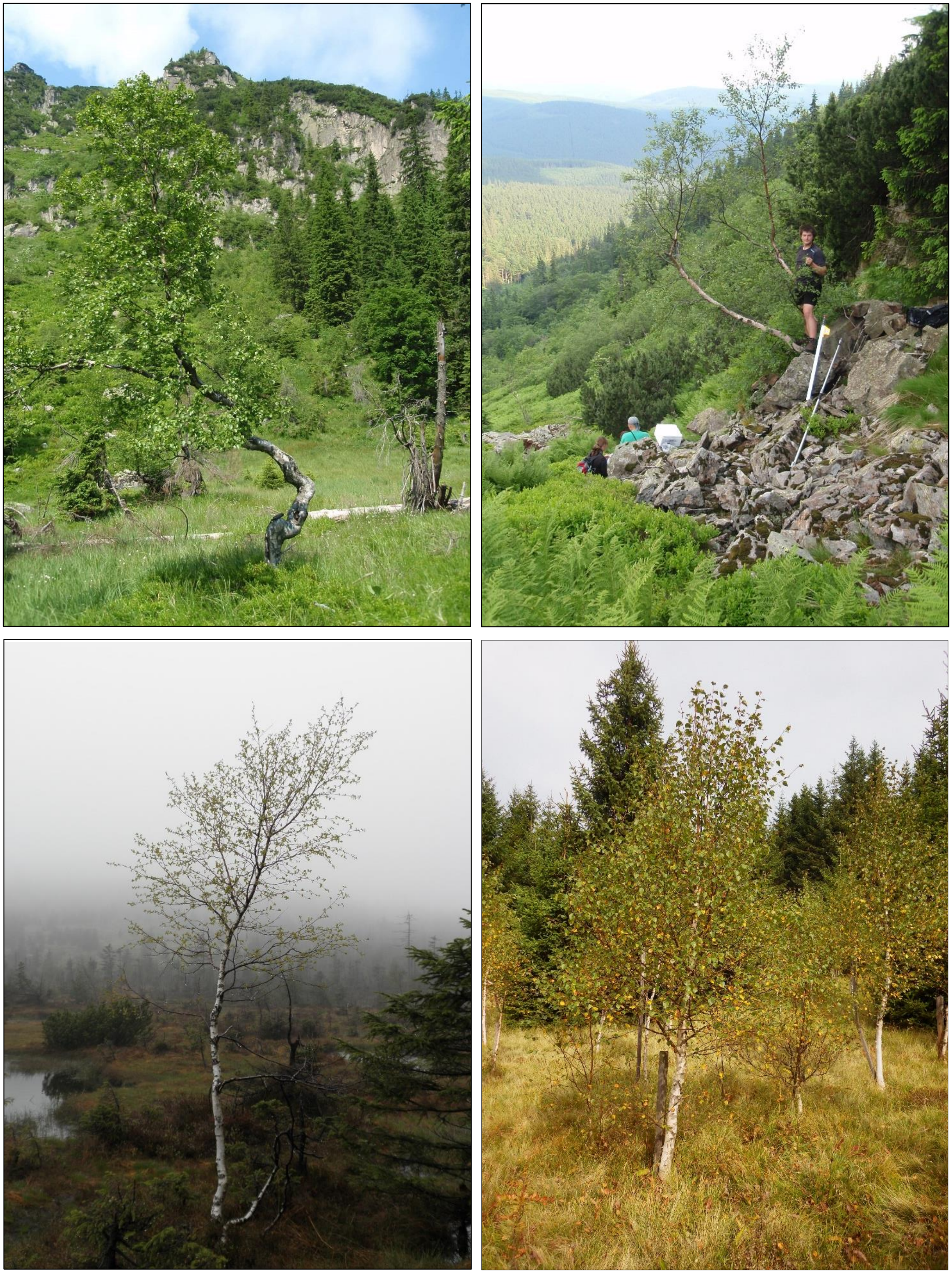

S2 Fig. ***Betula carpatica.***

Upper left: an individual at the bottom of a glacial carved valley in the Krkonoše Mts. Photo by Kuneš (2010).
Upper right: an individual at an avalanche slope of a corrie in the Krkonoše Mts. Photo by Kuneš (2010).
Lower left: an individual on a mountain peat bog in the Jizerské hory Mts. Photo by Baláš (2010).
Lower right: an individual in the experimental plantation in the Jizerské hory Mts. Photo by Kuneš (2018).
