# Supplementary figures and images for: Is Betula carpatica genetically distinctive? A morphometric, cytometric and molecular study of birches in the Bohemian Massif with a focus on Carpathian birch
Source: PLoS One. 2019 Oct 31;14(10):e0224387. doi: 10.1371/journal.pone.0224387 (PMC6822711; doi:10.1371/journal.pone.0224387)

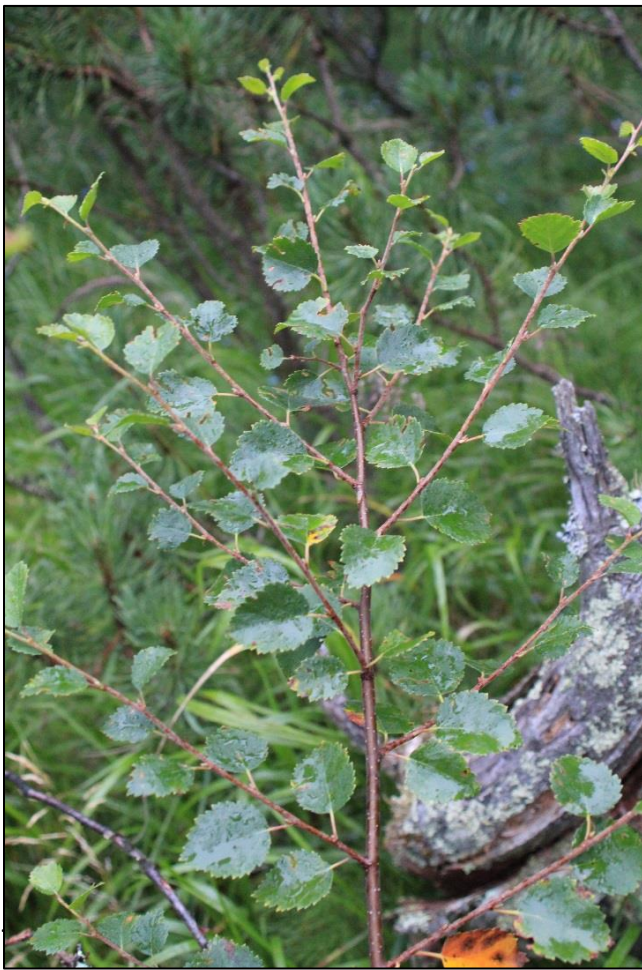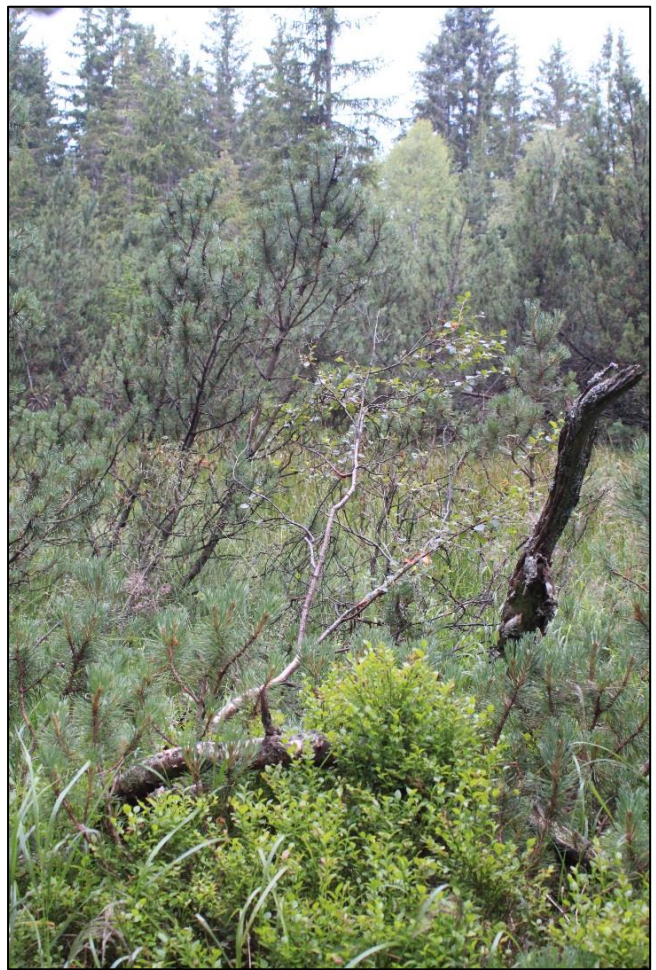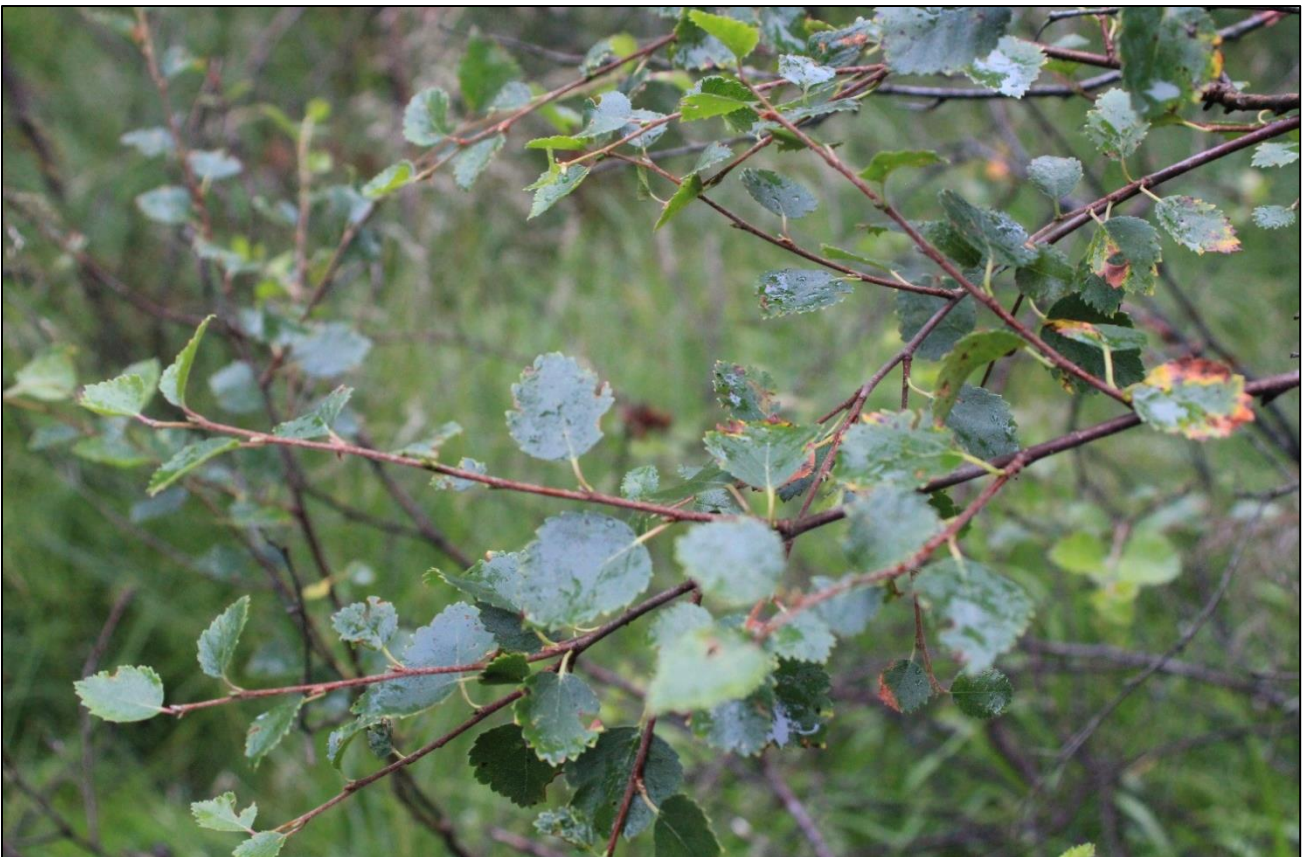

**S3 Fig.** *Betula*  $\times$  *seideliana* sensu Missbach 1908.  
Photos taken by Pavličko (2012) in the Šumava Mts.

Supplement: S3 Fig — Photos taken by Pavličko (2012) in the Šumava Mts. (PDF) [file pone.0224387.s003.pdf]
